# Histidine containing dipeptides protect epithelial and endothelial cell barriers from methylglyoxal induced injury

**DOI:** 10.1038/s41598-024-77891-9

**Published:** 2024-11-04

**Authors:** Charlotte Wetzel, Nadia Gallenstein, Verena Peters, Thomas Fleming, Iva Marinovic, Alea Bodenschatz, Zhiwei Du, Katharina Küper, Clelia Dallanoce, Giancarlo Aldini, Thomas Schmoch, Thorsten Brenner, Markus Alexander Weigand, Sotirios G. Zarogiannis, Claus Peter Schmitt, Maria Bartosova

**Affiliations:** 1 Centre for Paediatric and Adolescent Medicine, Medical Faculty Heidelberg, Heidelberg University, Heidelberg, Germany; 2 Department of Anesthesiology, Medical Faculty Heidelberg, Heidelberg University, Heidelberg, Germany; 3 Internal Medicine I and Clinical Chemistry, Medical Faculty Heidelberg, Heidelberg University, Heidelberg, Germany; 4 German Center for Diabetes Research (DZD), Neuherberg, Germany; 5 Department of Pharmaceutical Sciences, Medicinal Chemistry Section “Pietro Pratesi”, University of Milan, Milan, Italy; 6 Department of Anesthesiology and Intensive Care Medicine, Hôpitaux Robert Schuman – Hôpital Kirchberg, Luxembourg City, Luxembourg; 7 Department of Anesthesiology and Intensive Care Medicine, University Hospital Essen, University Duisburg-Essen, Essen, Germany; 8 Department of Physiology, Faculty of Medicine, School of Health Sciences, University of Thessaly, Larissa, Greece; 9 Division of Pediatric Nephrology, Center for Pediatric and Adolescent Medicine, Im Neuenheimer Feld 430, 69120 Heidelberg, Germany

**Keywords:** Dipeptides, Carbonyl stress, Barrier integrity, Transepithelial resistance, Ionic permeability, Zonula-occludens, Drug discovery, Physiology, Medical research, Molecular medicine

## Abstract

Integrity of epithelial and endothelial cell barriers is of critical importance for health, barrier disruption is a hallmark of numerous diseases, of which many are driven by carbonyl stressors such as methylglyoxal (MG). Carnosine and anserine exert some MG-quenching activity, but the impact of these and of other histidine containing dipeptides on cell barrier integrity has not been explored in detail. In human proximal tubular (HK-2) and umbilical vein endothelial (HUVEC) cells, exposure to 200 µM MG decreased transepithelial resistance (TER), i.e. increased ionic permeability and permeability for 4-, 10- and 70-kDa dextran, membrane zonula occludens (ZO-1) abundance was reduced, methylglyoxal 5-hydro-5-methylimidazolones (MG-H1) formation was increased. Carnosine, balenine (ß-ala-1methyl-histidine) and anserine (ß-ala-3-methyl-histidine) ameliorated MG-induced reduction of TER in both cell types. Incubation with histidine, 1-/3-methylhistidine, but not with ß-alanine alone, restored TER, although to a lower extent than the corresponding dipeptides. Carnosine and anserine normalized transport and membrane ZO-1 abundance. Aminoguanidine, a well-described MG-quencher, did not mitigate MG-induced loss of TER. Our results show that the effects of the dipeptides on epithelial and endothelial resistance and junction function depend on the methylation status of histidine and are not exclusively explained by their quenching activity.

## Introduction

Integrity of epithelial and endothelial barriers are crucial for the health; their disruption mediates numerous disease states, such as sepsis, diabetes, cancers, inflammatory bowel disease^1^, resulting in impairment of the barrier integrity, associated with hyperpermeability and dysregulation of paracellular transport. Paracellular transport depends on the composition of tight junctions, mainly claudin proteins, which are localized between adjacent epithelial and endothelial cells and connected to the actin cytoskeleton via Zonula occludens^2^.

An important mediator of barrier breakdown is the reactive dicarbonyl methylglyoxal (MG)^3^. MG derives mainly from the spontaneous fragmentation of byproducts of glycolysis, via the degradation of acetone and threonine or can originate from the degradation of Amadori products^4,5^. MG leads to the formation of advanced glycation endproducts (AGE), a heterogeneous group of molecules formed through the Maillard reaction, in which reducing sugars glycate protein amino groups^6^. MG and MG-derived AGE induce endothelial dysfunction via a variety of mechanism, i.e. inducing oxidative stress, inflammation, apoptosis, and endoplasmic reticulum stress^3,7–9^ and are associated with the onset and progression of numerous pathologies including diabetes, cancer, and liver and kidney disease^10–13^.

Carnosine (ß-alanine-histidine, Carn) and anserine (β-alanyl-3-methylhistidine, Ans), scavenge various aldehydes from lipid and sugar oxidation, with α-unsaturated aldehydes^14^. Carnosine prevented MG-induced formation of AGE in cells under metabolic stress^15^ and in diabetic mice^16–20^. In cell-free systems, carnosine catalyzes the formation of MG oligo/polymeric products^21^ but in vivo, the mechanism of action is unlikely to be through intracellular quenching^21,22^. Carnosine may also act indirectly by activating endogenous anti-carbonylation systems, e.g. glyoxalase 1 or via the Nrf2 pathway^22^. Research on the properties of balenine is less extensive, but it has been found that balenine has a higher antioxidant and iron-chelating capacity than carnosine and anserine^23^. In contrast, balenine quenches reactive aldehydes such as 4-hydroxy-2-nonenal less effectively^24^ and is more resistant to degradation by carnosinase 1 (CN1), resulting in better bioavailability in vivo^25^.

Considering the well-known pathogenic role of the α-unsaturated carbonyl MG, we investigated whether histidine-dipeptides can ameliorate MG-induced disruption of endothelial and epithelial integrity in human endothelial cells (HUVEC) and in human proximal tubular cells (HK-2). Since the imidazole ring of histidine is highly reactive^26^, we additionally investigated whether methylation of histidine effects MG-induced AGE formation by measuring hydroimidazolone isomer 1 (MG-H1)^21^ and the endothelial and epithelial integrity of the membrane characterized by measuring transepithelial resistance (TER), membrane permeability and the membrane abundance of zonula occludens-1, an adaptor protein connecting tight junctions to the actin cytoskeleton, thus ensuring the membrane integrity^27^.

We compared the effects of anserine (consisting of ß-alanine and 3-methylhistidine) and balenine (ß-alanine and 1-methylhistidine) with carnosine (ß-alanine and histidine) and the corresponding components of the dipeptides on MG-induced MG-H1 formation and protection of the barrier integrity. Reducing carbonyl stress and associated pathophysiology is a potential strategy to prevent disease.

## Results

### Methylglyoxal-induced loss of transepithelial and transendothelial resistance is rescued to different extent by histidine containing dipeptides

**Fig. 1:**
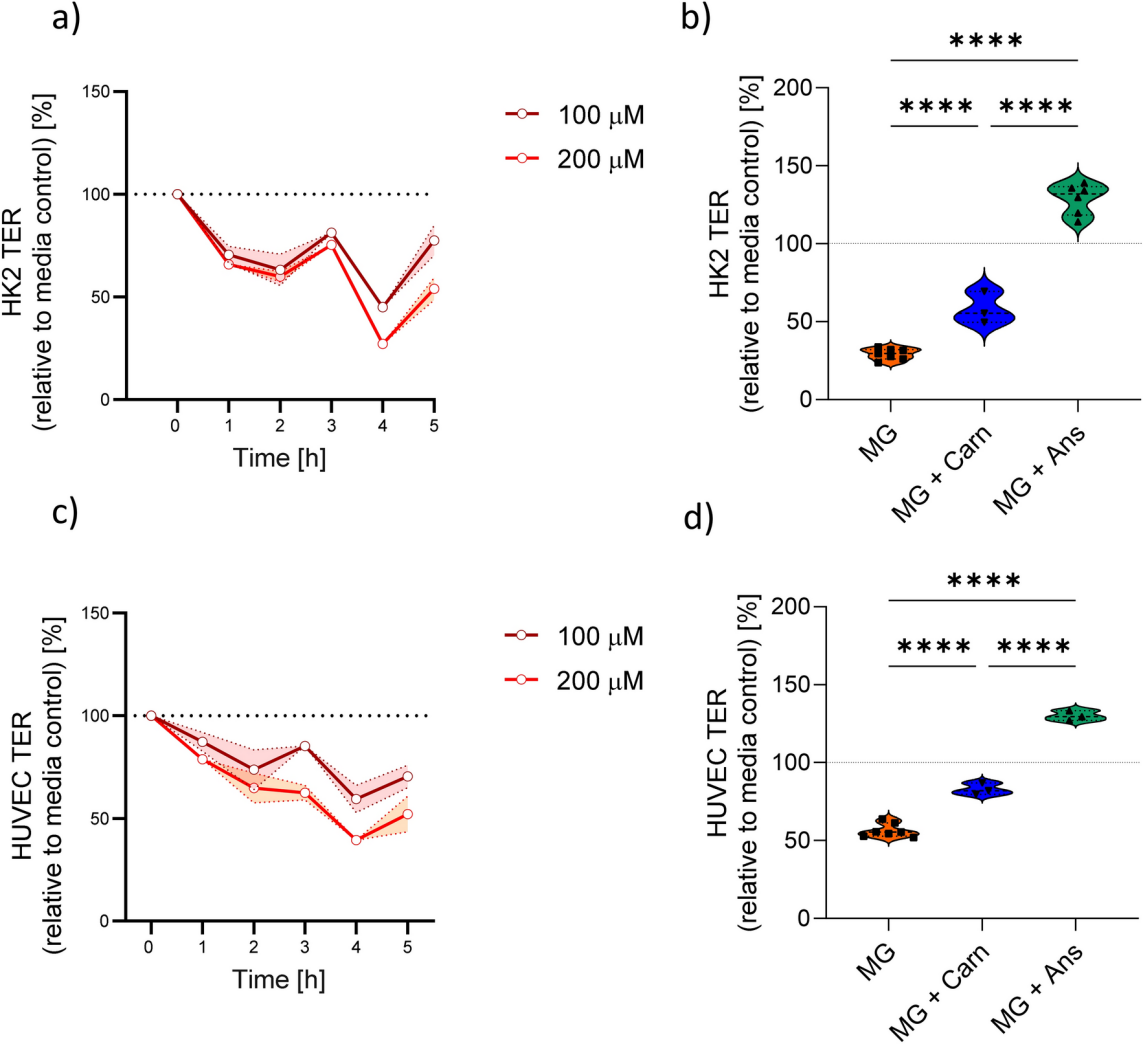
Transepithelial and transendothelial resistance (TER). TER decreased dose-dependently upon exposure to methylglyoxal (MG, 200 µM) in human proximal tubulus cell (HK2, **a**) and human umbilical vein endothelial cells (HUVEC, **c**). The decrease of TER was prevented by co-incubation with carnosine (Carn) and anserine (Ans, both 70 mM) in both cell types (**b**,**d**). One-way ANOVA with Tukey´s test and correction for multiple comparison. ****p < 0.0001.

Incubation of human proximal tubular (HK2) cells with methylglyoxal (MG) over 5 h decreased transepithelial resistance dose-dependently (two-way ANOVA < 0.0001, Fig. 1a). Transepithelial resistance (TER) decrease induced by MG (200 µM) was ameliorated by co-incubation with 70 mM carnosine (Carn). Anserine (Ans) in the same concentration not only reversed MG-induced TER loss, but increased TER above control level (129 ± 39.8% in comparison to media control, p < 0.0001) in HK2 cells (Fig. 1b), this effect was dose-dependent and was not observed when Ans was added to HK2 cells. In human umbilical vein endothelial cells (HUVEC), 70 mM Ans led to an increase in TER when added to the cell media (Fig. S1). Notably, while endothelial cell viability, assessed by the MTT assay, was reduced by Carn, it remained unchanged with Ans, even at high concentrations (Fig. S2). Transendothelial resistance was halved after exposure to 200 µM MG (Fig. 1c). This reduction was prevented by both Ans and Car, but co-incubation with Ans elevated TER values above control levels (130 ± 3.2% vs. control, p < 0.0001, Fig. 1d). Next, the protective impact of other histidine containing dipeptides, methylated at different positions was analyzed. While all histidine-containing dipeptides mitigated the MG-induced TER reduction, only those containing 3-methylhistidine were able to fully restore TER to control levels in both epithelial and endothelial cells (Table 1).

**Table 1:** Different histidine containing dipeptides (70 mM, unless stated otherwise) were used to rescue MG-induced TER decrease (200 µM MG).

	HK-2		p-value (vs. MG)	HUVEC		p-value (vs. MG)
	TER (% control)	p-value (vs. control)		TER (% control)	p-value (vs. control)	
Methylglyoxal	29 ± 3.6	< 0.0001	n.a	56 ± 4.4	< 0.0001	n.a
Carnosine	58 ± 10.2	< 0.0001	< 0.0001	83 ± 3.7	< 0.0001	< 0.0001
Anserine	129 ± 39.8	< 0.0001	< 0.0001	130 ± 3.2	< 0.0001	< 0.0001
Balenine	n.m	n.m	n.m	104 ± 5.3	0.87	< 0.0001
L-Histidine	58 ± 17.7	< 0.0001	< 0.0001	65 ± 4.1	< 0.0001	0.011
3-Methyl-Histidine	96 ± 5.7	0.994	< 0.0001	103 ± 2.8	0.99	< 0.0001
1-Methyl-Histidine	51 ± 7.1	< 0.0001	0.0004	84 ± 2.7	< 0.0001	< 0.0001
ß-Alanine	28 ± 1.2	< 0.0001	0.186	60 ± 0.98	< 0.0001	0.865
Methyl-Alanine	41 ± 2.4	< 0.0001	0.99	62 ± 3.9	< 0.0001	0.124
ß-Alanine + 3-Methyl-Histidine*	76 ± 2.7	< 0.0001	< 0.0001	75 ± 0.51	< 0.0001	< 0.0001

*HK-2* human proximal tubulus cells; *HUVEC* human umbilical vein endothelial cells; *TER* transepithelial resistance; *MG* methylglyoxal.

While almost all of them prevented MG induced barrier disruption after 5 h, only 3-methylhistidine had the capacity to keep TER at medium control levels.

*35 mM + 35 mM.

### Paracellular transport is higher with MG and cell-type dependently normalized with carnosin and anserine

**Fig. 2:**
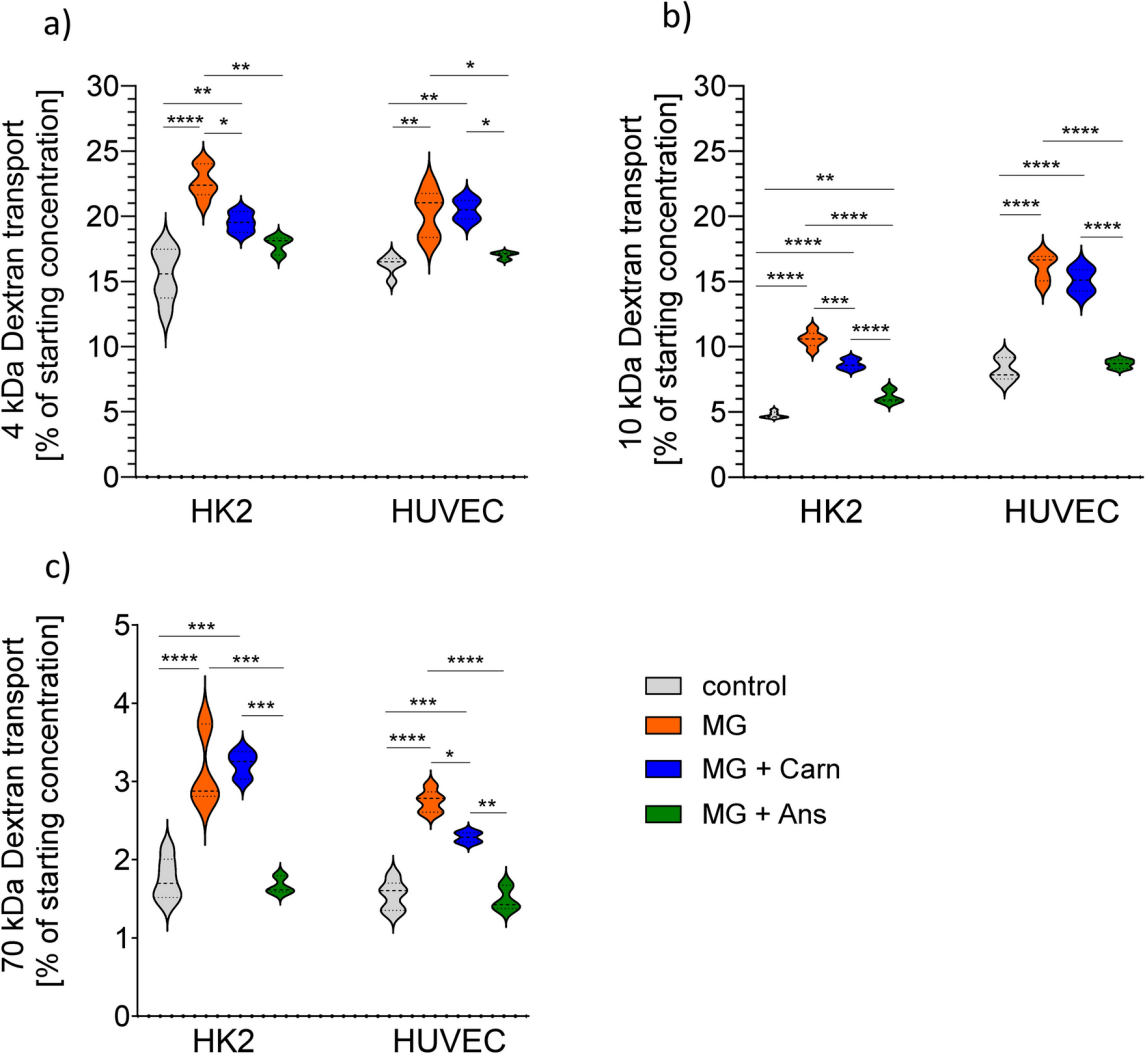
Paracellular transport capacity for dextrans of different molecular sizes in epithelial and endothelial cells. Paracellular transport of 4 kDa (**a**), 10 kDa (**b**) and 70 kDa (**c**) fluorescein labelled dextrans across human proximal tubulus (HK2) and human umbilical vein endothelial (HUVEC) monolayers increased after 5 h of exposure to methylglyoxal (MG, 200 µM). This increase could be partly prevented by co-incubation with carnosine (Carn), anserine (Ans, both 70 mM) treated cells were comparable to medium treated controls. Two-way ANOVA followed by Dunnett’s test corrected for multiple comparisons. *p < 0.05, **p < 0.01, ***p < 0.001, ****p < 0.0001.

In line with the impaired transepithelial and transendothelial resistance, paracellular transport for 4-, 10- and 70-kDa FITC labeled dextrans was higher upon MG exposure in both cell lines. After 4 h, transport rate through HK2 monolayer increased by 50% for 4-kDa and 100% for 10-kDa and 70-kDa dextran respectively (all p < 0.0001, Fig. 2a–c). After anserine and carnosine co-incubation, transport of 4-kDa and 10-kDa dextrans was comparable to the medium control, but only anserine and not carnosine reduced 70-kDa transport rate (Fig. 2c). In endothelial cells, MG induced similar effects inducing barrier disintegration which was preventable by co-incubation with carnosine but to a much smaller extent compared to when cells were incubated with anserine. Baseline transport rates for dextrans in HUVEC were comparable with HK2 cells. In contrast to HK-2, carnosine did not prevent MG induced endothelial hyperpermeability for 4- and 10-kDa dextrans in HUVEC (Fig. 2a,b).

### Response of ZO-1 at the cell membrane to MG with carnosine and anserine differs in HK2 and HUVEC

**Fig. 3:**
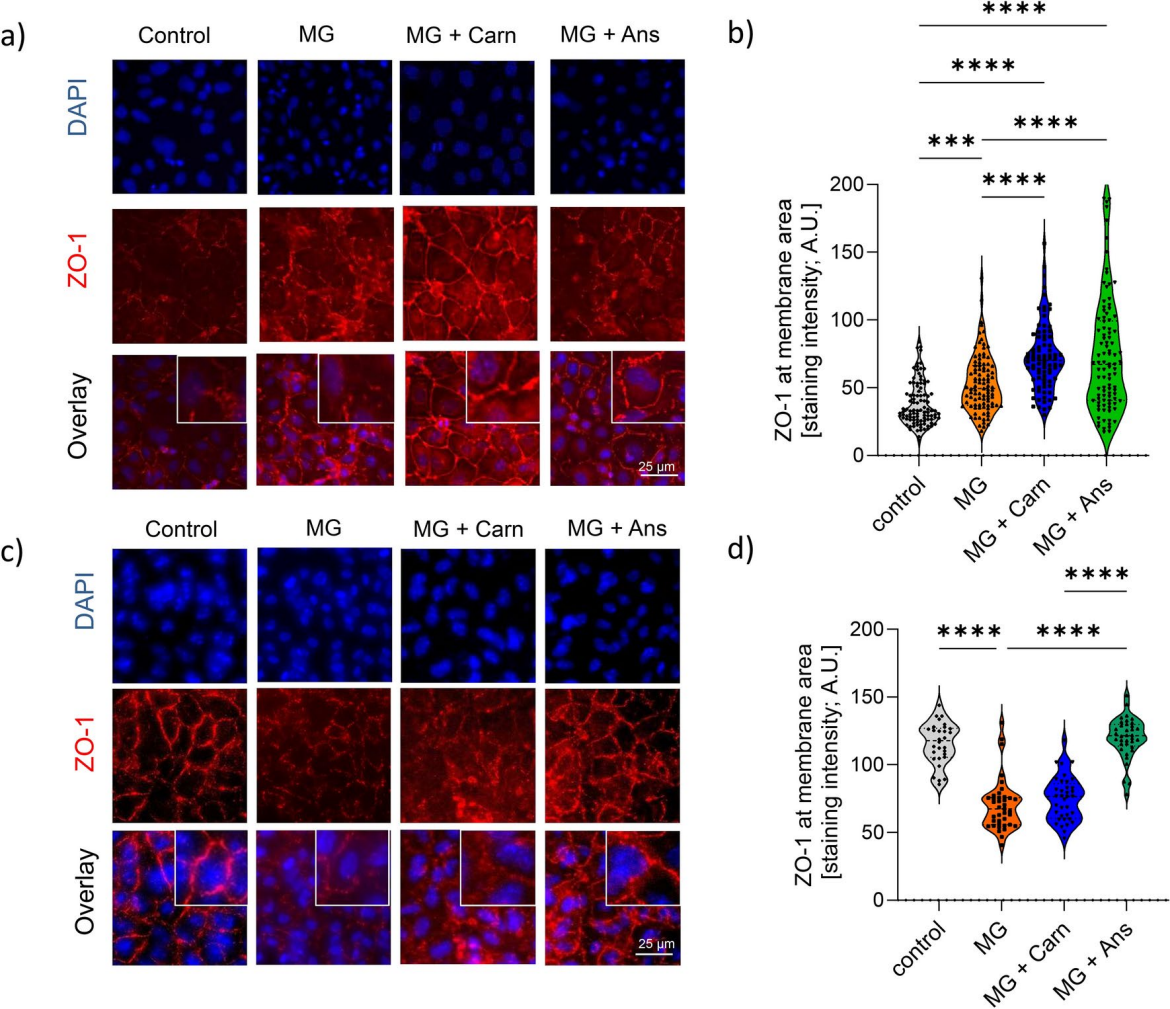
Zonula occludens (ZO-1) distribution in cell membranes of epithelial and endothelial cells. Methylglyoxal (MG, 200 µM) induces cell type specific ZO-1 cell membrane distribution. Representative immunostaining of ZO-1 showing the pattern at cellular membrane areas followed by quantification of membrane ZO-1 in polarized HK 2 (**a**,**b**) and HUVEC (**c**,**d**) grown on transwell filters upon exposure to MG and treatment with carnosine (Carn) and anserine (Ans, both 70 mM). MG resulted in discontinuous ZO-1 membrane distribution, this effect was prevented by Ans treatment. One-way ANOVA with Tukey´s test corrected for multiple comparisons. ***p < 0.001, ****p < 0.0001.

ZO-1 pattern at cell membranes was visualized and quantified in HK2 and HUVEC cells. In HK2 incubated by cell medium the ZO-1 at the membrane was low, but continuous. Upon MG exposure, the staining intensity increased, but showed discontinuous pattern. Carnosine and anserine improved the ZO-1 and increased the abundance. (Fig. 3a,b). In HUVEC, ZO-1 was discontinuous with low intensity after exposure to MG. Anserine, but not carnosine prevented ZO-1 dissociation (Fig. 3c,d).

### MG-H1 quenching capacity of anserine and carnosine

**Fig. 4:**
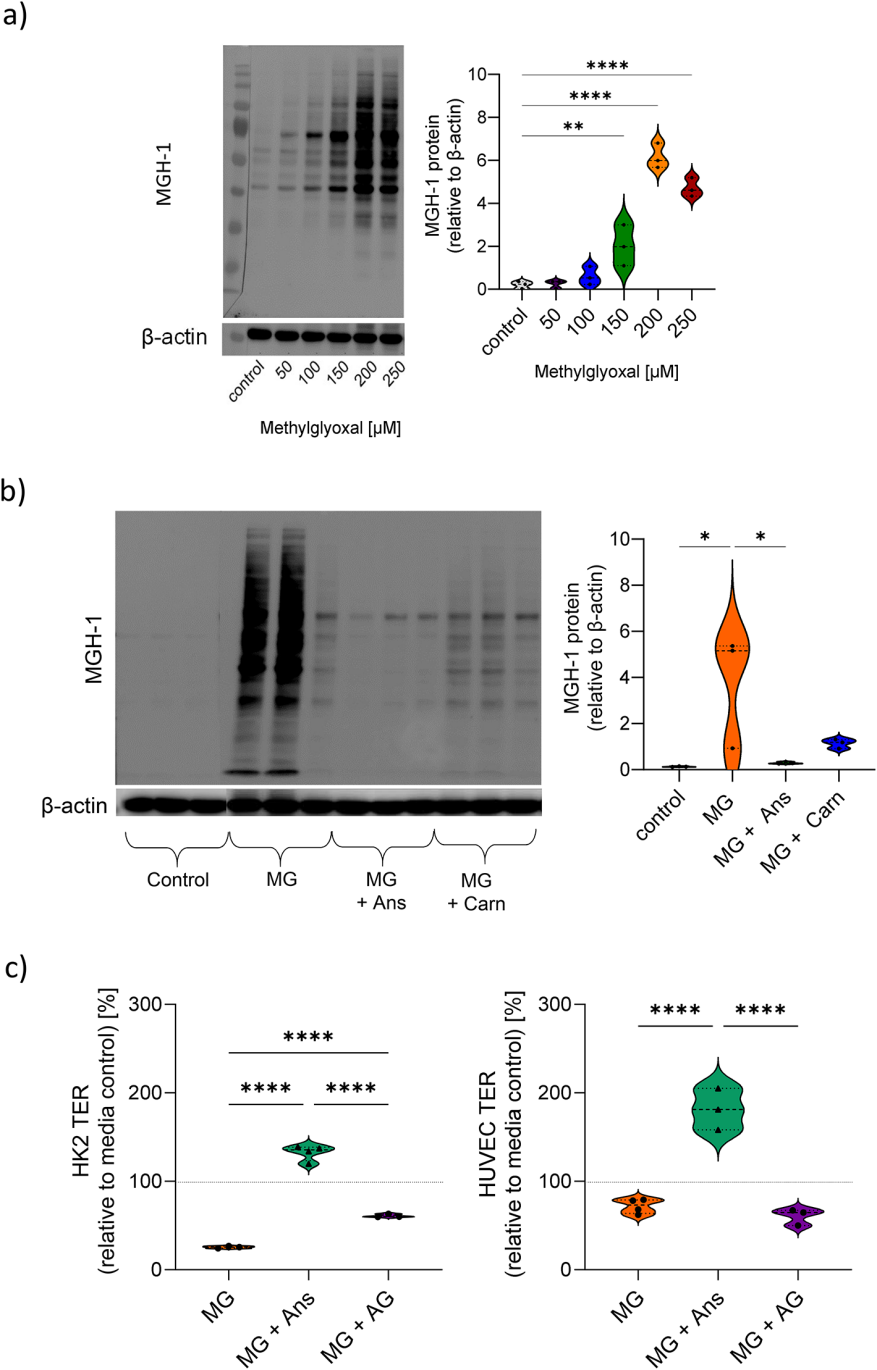
Formation and quenching of the methylglyoxal 5-hydro-5-methylimidazolone (MG-H1). Methylglyoxal (MG) induces formation of the MG-H1 after 5 h of exposure in HK2 cells. Anserin (Ans) and carnosine (Carn, both 70 mM) quench MG-H1 in HK2. Representative western blot image and quantification of MG-H1 relative to ß-actin in HK2 cells after exposure to MG (**a**) and the reduction of MG-H1 after co-incubation with Ans and Carn (**b**). Effect of well described MG-H1 quencher aminoguanidine (AG) on transepithelial and transendothelial resistance (**c**). While in HK-2 cells (left) AG resulted in higher TER compared to MG treatment only, this effect was significantly lower compared to Ans. In HUVEC (right), AG had no effects. One-way ANOVA followed by Tukey’s test with correction for multiple comparisons. *p < 0.05, ****p < 0.0001.

MG dose dependently increased MG-H1 formation up to sixfold relative to control level (p < 0.0001) within five hours (Fig. 4a) in HK2. MG-H1-formation was partly prevented by addition of anserine and carnosine (Fig. 4b) and correlated with TER loss (R^2^ = 0.48, p = 0.036). In endothelial cells, in-cell western blot was used to compare the quenching capacity of carnosine and anserine with aminoguanidine (AG), a well-established MG quencher. While AG quenched MG very efficiently (99 ± 0.5%, p < 0.0001 vs. MG, MG-H1 signal was not detectable), no quenching activity was detectable for anserine and carnosine at the same concentration (Fig. S3). When higher concentrations of anserine and carnosine were used, MG could be quenched to some extend (32 ± 14 and 32 ± 15% of total MGH-1 for anserine and carnosine respectively; both < 0.0001 vs. MG). We next studied protective effect of AG on barrier integrity. TER loss was less pronounced with MG + AG compared to MG only, but the protective capacity of AG did not reach anserine levels (Fig. 4c) in HK2. In HUVEC, AG did not prevent MG-induced TER loss (Fig. 4c).

## Discussion

Barrier integrity of polarized cells is vital for physiological functions of tissues^28^. Tight junctions together with their adaptor proteins ensure proper barrier function and control paracellular transport in epithelial and endothelial cells^29^. Carbonyl stress induces cellular dysfunction through the formation of advanced glycation endproducts (AGE)^30,31^, MG-H1 is enriched in tissues exposed to carbonyl stress. Methylglyoxal induced loss of barrier function of renal tubular epithelial and human umbilical vein endothelial cells, both exhibited lower transepithelial and transendothelial resistance and accordingly paracellular transport was higher for molecules of small, middle and large molecules. The potential of dipeptides in preventing barrier function loss has been demonstrated for Alanyl-Glutamine in endothelial cells exposed to glucose and glucose degradation product containing peritoneal dialysis solutions^32^. The histidine containing dipeptides carnosine and anserine can prevent MG-induced AGE and carboxyethyllysine (CEL) formation in vitro^21^.

We now demonstrate, that carnosine and anserine ameliorated MG-reduced epithelial and endothelial resistance loss, a protective action beyond their quenching activity. Anserine proved to be more effective than carnosine, followed by balenine which was able to rescue MG-induced transendothelial resistance loss more effectively then carnosine, but not as effectively as anserine. While balenine normalized MG- reduced TER, anserine resulted in even higher resistance as compared to the medium control level in both cell types. When anserine was supplemented to medium only, TER increased in HUVEC, but not in HK2, suggesting beneficial effects beyond MG quenching. In HUVEC, anserine added to cell media resulted in higher resistance of the endothelial barrier. These data suggest, that anserine activates (cell)- specific protective pathways resulting in the sealing of the cell monolayer. The major histidine derivates present in the human body are 3-methylhistidine and 1-methylhistidine which arise from protein catabolism or anserine metabolism^33^, but their properties in comparison to histidine without methyl groups remain unclear. Protection of epithelial and endothelial cells from carbonyl induced barrier disruption was exerted mainly by 3-methylhistidine, which was more effective than 1-methylhistidine, but both were far more effective than histidine alone. Single compounds, ß-alanine and methyl-alanine had no beneficial effect on MG-induced TER disruption. ß-alanine in combination with 3-methylhistidine restored TER function in HK-2 and HUVEC, but again did not reach the values of anserine. These findings are in line with randomized controlled studies in critically ill patients showing beneficial effects of alanyl-glutamine^34^, while glutamine was less efficient^35^. There is evidence from plant studies that dipeptide specific effects are mediated by protein binding and regulation of protein- (mis-) folding^36–38^. Slight modifications of dipeptides (e.g. diphenylalanine) alter the molecular design of peptide-based nanostructures^39^. Thus, it is conceivable that dipeptides exert specific actions which are beyond their function as amino acid donors.

Following the transepithelial and transendothelial resistance studies, we analyzed paracellular transport using three different dextrans. MG induced hyperpermeability in epithelial and endothelial cells. Similar actions have been observed in diabetic complications as retinal capillary endothelial cell junction disruption^8^, MG-H1 disrupted tight junctions in intestinal cells^40^.

Anserine effectively reduced MG-induced transport in both cell lines for all three dextran molecules. Carnosine on the other hand, was far less effective, only significantly reducing 70-kDa dextran transport in HUVEC and 4-kDa dextran transport in HK-2. In line with these findings, anserine but not carnosine induced higher and more organized abundance of ZO-1. Dissociation of ZO-1 is associated with carbonyl stress in endothelial cells in vitro^41^ and in human tissues^42^ exposed to carbonyl stress. Of note, while the effects of carnosine and anserine on ZO-1 were similar in epithelial and endothelial cell lines, ZO-1 abundance under control medium conditions was low in HK-2 cells and increased upon MG treatment. In kidney, the paracellular permeability decreases from the proximal tubule to the collecting duct, accompanied by increasing abundance of ZO-1 and occludin and varying expression levels of tight junction components^43^. HK-2 are derived from proximal tubules, ZO-1 expression under quiescent conditions was low. In HUVEC, the ZO-1 intensity was lower after exposure to MG. In both cell types, incubation with anserine and carnosine increased abundance of the scaffolding protein ZO-1, suggesting higher tight junction network integrity and stability, which again supports the finding of an enhanced barrier. Again, the effect of anserine was much more pronounced compared to carnosine. Stabilizing actions of carnosine on ZO-1 have been described in human retinal pigment epithelial cells and retinal capillary endothelial cells of patients with age-related macular degeneration^44^ and in rat blood brain barrier^44,45^, anserine has not been studied.

To investigate whether the protective effects of anserine and to a smaller extend of carnosine on epithelial and endothelial function can be explained by MG-quenching, we compared the quenching efficacy of the two dipeptides with the known MG-quencher aminoguanidine^46^. Previous studies showed that while carnosine and anserine can also scavenge MG, very high doses^21,47^ are required and the efficacy is far below of aminoguanidine^48^. Despite the better quenching property of aminoguanidine, its effect on TER was significantly lower than that of anserine. This could be due to the fact that the high protective effect of anserine is not only explained by the quenching property of MG, but also by other mechanisms, such as binding to endothelial membrane proteins. Anserine and carnosine exhibit antioxidant properties^49,50^ and promote H_2_S formation in vitro and ex vivo^51^, the latter via induction of heat shock protein-70. In addition, aminoguanidine has toxic effects reducing TER. In line with this, in animal models of diabetes, aminoguanidine lowered AGE formation and prevented nephropathy^52,53^, retinopathy^54^ and neuropathy^55^, clinical trials, however, had to be discontinued due to adverse events^56,57^.

Our findings demonstrate a strong protective effect of anserine against carbonyl stress in human proximal tubular and endothelial cells and for the first time identify the importance of the methylation of the histidine dipeptides. While MG-quenching and membrane ZO-1 upregulation in part explain our findings, further mechanistic studies are required. Anserine and alanyl-glutamine have been shown to upregulate heat shock response^49,58–60^. The concentrations of MG, of the dipeptides and of aminoguanidine used in our in vitro models exceed the concentrations observed in patients, e.g. with diabetes mellitus^61^. These, however, are often needed in in vitro cell systems, especially if strong MG specific effects are studied^21,62^, as which we assessed in the present study by the MG-H1 formation rate. While anserine did not exhibit any signs of cell toxicity, the high aminoguanidine concentrations may exert significant toxicity possibly explaining the low efficacy in TER rescue as compared to the dipeptides, despite efficient MG quenching.

## Conclusions

We describe protective actions of carnosine and of its methylated form anserine from carbonyl stress induced epithelial and endothelial barrier disintegration and dysfunction with anserine being more effective than carnosine. Methylation of the histidine at specific sites confers the effective protective action against MG induced injury. Specific dipeptides may therefore represent an innovative therapeutic approach for diseases associated with epithelial and endothelial barrier dysfunction.

## Materials and methods

### Cell culture

Primary cultures of human endothelial umbilical vein cells (HUVEC) from pooled donors were purchased commercially (Promocell, Heidelberg, Germany). They were kept in endothelial cell growth medium supplemented with growth factors (Supplement Mix, Promocell, Heidelberg, Germany) and 1% penicillin and streptomycin (v/v). Immortalized human proximal tubular epithelial cells (HK-2, American Type Culture Collection CRL-2190, Manassas, VA, USA) were cultured in RPMI 1640 GlutaMAX medium (Thermo Fisher Scientific, Waltham, MA) with 0.1 or 10% fetal calf serum (v/v) and 1% penicillin and streptomycin (v/v) at 37 °C with 5% CO_2_ and were used until P15. Both cell lines were kept under standard conditions at 37 °C and 5% CO_2_.Cells were split using 0.25% EDTA trypsin (Thermo Fisher Scientific, Waltham, USA).

### Transepithelial resistance measurements

5 × 10^4^ cells (HUVEC) and 2 × 10^4^ cells (HK-2) were seeded on transwell inserts (Sarstedt, Nümbrecht, Germany) in 24-well plates. Transepithelial electrical resistance (TER) was used as a marker to determine the integrity of the cell monolayer and was assessed by using EVOM device and a silver/silver-chloride chopstick electrode (STX2) (World Precision Instruments, Hertfordshire, UK). Resistance was calculated after subtraction of blank values (filters without cells) in Ohm.cm^2^ after multiplication with the filter area (0.33 cm^2^) for standardization.

TER of the cell monolayer was measured every day until reaching a plateau (day 4–5 after seeding for HUVEC, day 3–4 for HK-2), media was exchanged every 2 to 3 days. After reaching a plateau, cells were incubated with treatment compounds, cells with medium served as controls. TER was measured every hour for 5 h.

### Methylglyoxal exposure model

HUVEC and HK-2 cells were used in experiments after reaching confluence as monitored by TER measurements. Methylglyoxal (MG, Sigma-Aldrich, UK) stock of 825 µM was prepared in sterile water and kept in dark on ice during all experiments. MG was diluted in the cell culture media and cells were incubated with indicated concentrations. Since MG is metabolized fast, it was renewed every two hours in the given concentrations in apical compartment of every well to maintain carbonyl-stress.

### Dipeptides and single amino acids

All dipeptides (DP) were purchased commercially except for balenine, which was prepared according to a synthetic procedure recently described^24^. DP were diluted in cell media in indicated concentrations. In a subset of experiments, low MG concentrations were used with the same molar ratio of DP. Single amino acids were mixed in concentrations corresponding to the DP concentration. Rescue treatments were given as single dose at starting timepoint and were not renewed unless otherwise stated.

### Molecular size dependent transport experiments

Experiments were performed in transwell system as described previously^63^. After one-hour exposure to the treatment solution, the treatment solution was exchanged and 1 mg/ml of fluorescein isothiocyanate (FITC) dextran was added to the apical compartment. Unlabeled dextran of the same molecular weight and concentration was added to the basolateral compartment to prevent gradient driven transport. After five hours, samples were taken from the lower compartment and fluorescence was measured by fluorometer (Tecan, Männedorf, Switzerland). Data are presented as % of dextran which was transported from the apical to the basolateral compartment.

### Western blot and in-cell western blot

RIPA-buffer (radio-immunoprecipitation assay buffer: 150 mM NaCl, 0.1% Triton X-100, 0.5% sodium deoxycholate, 0.1% SDS, 50 mM Tris–HCl; pH 8.0) and protease inhibitor (cOmplete tablets, Mini EASYpack, Roche Diagnostics, Mannheim, Germany) were used to lyse cell samples and 20 µg of total protein samples were separated by SDS-PAGE in 10% polyacrylamide gels. Samples were transferred to a nitrocellulose membrane by semi-dry blot. The membrane was then blocked with 3% goat serum in non-protein blocking buffer (Thermo Fisher Scientific, Waltham, USA) as bovine serum albumin (BSA) and milk-powder contain AGEs and will therefore compete for the antibody. After blocking, the membranes were incubated with the primary MG-H1 antibody (10 mg/ml in non-protein blocking buffer) overnight, shaking at 4 °C. After washing with Tris-buffered saline with Tween 20 (TBS-T), the membrane was incubated with a secondary horseradish peroxidase (HRP)-conjugated antibody (goat-anti-rat, 1:1000 in non-protein blocking buffer, Cell Signaling) for 1 h at room temperature (RT). Protein expression of target protein was normalized to β-Actin expression. Western blots were developed with Clarity Western enhanced chemiluminescence (ECL) Substrate (BioRAD, Hercules, CA) according the manufacturer’s protocol, imaged via a fluorescence imaging system (PEQLAB fusion, PEQLAB, Erlangen, Germany) and quantified via ImageJ^64^.

In-cell western blot^65^ was used to quantify MG-quenching capacity of histidine containing dipeptides in HUVEC. 30.000 cells per well were seeded in a 96-well, black body, clear bottom plate with square wells and allowed to adhere overnight. After treatment, cells were fixed with 4% paraformaldehyde (PFA) in phosphate buffered saline (PBS) overnight at 4 °C. On the next day the cells were washed two times with PBS, permeabilized with 0.1% Triton X100 in TBS (15 min at RT) and blocked with 3% goat serum in TBS for 1 h at RT. After incubation with the MG-H1-x-biotin antibody (produced in house^66^,) overnight at 4 °C (2 µg/ml in 3% goat serum in TBS, the cells were washed three times with TBS + 0.1% Tween 20 and IRDye® 800CW Streptavidin was added (LICOR, 1:1000 in 3% goat serum in TBST; 1 h protected from the light). After washing with TBST, CellTag™ 700 Stain was added (LICOR; 1:500 in TBST, 100 µl/well; 1 h protected from the light). Finally, after three more washing steps with TBST and three washing steps with TBS the plate was scanned with Odyssey® IR scanner, and analyzed using the Odyssey® imaging software.

### Immunostaining and imaging

Staining was performed from the same cell monolayers as TER and transport studies as established previously^63^. Briefly, transwell filters were washed, fixed with absolute ethanol and incubated with Alexa conjugated primary antibody against ZO-1 (1:1000, clone 1A12, ThermoFisher Scientific, Waltham, MA, USA). Filters were cut out and mounted on glass slides. Images were acquired on automated imaging machine (Acquifer, Heidelberg, Germany) in one run for all conditions. First, the whole filter area was imaged in 2 × magnification based on the DAPI staining. Next, 10 randomly selected areas were imaged with 20 × objective in DAPI and ZO-1 channel. Ten z-stacks with image distance of 3 µm were acquired. Analysis of the staining intensity was performed from grey scale ZO-1 images limited to the membrane areas by ImageJ^63^.

### Cell viability

MTT assay was used to measure endothelial cell viability. HUVEC were seeded on 96-well plates, allowed to attach and grow for 24 h. The same exposure model of MG ± Carn/Ans was applied as described above. At the end of the incubation period, 50 µl PBS (Thermo Fisher Scientific, Waltham, MA) with 2 mg/ml 3-(4, 5-dimethylthiazol-2-yl)-2, 5-diphenyltetrazolium bromide (MTT) was added to each well. This compound is converted to purple formazan crystals by metabolically active cells. After 4 h, the medium was discarded and cells were lysed with 200 µl dimethyl sulfoxide (DMSO) per well for 1 h. The absorption of the solution was measured spectrophotometrically at 590 nm, the absorption maximum of formazan crystals. Data are normalized to medium control (100%).

### Statistics

All experiments were performed independently at least three times in triplicates. Data are presented as mean ± standard deviation (SD), differences between the groups were tested by one-way ANOVA / two-way ANOVA followed by Tukey´s/Dunnett’s test corrected for multiple comparisons as appropriate. For in-cell western blot analysis, one-way ANOVA followed by Sidak´s multiple comparison correction was used in order to compare the quenching capacity of anserine and carnosine in with the quenching capacity of aminoguanidine, well described quencher serving as positive control. In all analysis two sided tests were used and p < 0.05 was considered significant. GraphPad Prism v9 (La Jolla, CA, USA) was used.

## Supplementary Information

Supplementary Information 1.

Supplementary Information 2.

## Data Availability

Supporting data is available from the corresponding author upon reasonable request.

## Abbreviations

AGE: Advanced glycation endproducts
AG: Aminoguanidine
Ans: Anserine
BSA: Bovine serum albumin
CEL: Carboxyethyllysine
CN1: Carnosinase 1
Carn: Carnosine
DMSO: Dimethyl sulfoxide
DP: Dipeptides
ECL: Enhanced chemiluminescence
FITC: Fluorescein isothiocyanate
HRP: Horseradish peroxidase
HK-2: Human proximal tubular cells
HUVEC: Human umbilical vein endothelial cells
MG: Methylglyoxal
MG-H1: Methylglyoxal 5-hydro-5-methylimidazolones
PFA: Paraformaldehyde
PBS: Phosphate buffered saline
RIPA-buffer: Radio-immunoprecipitation assay buffer
RT: Room temperature
SD: Standard deviation
TER: Transepithelial resistance
TBS-T: Tris-buffered saline with Tween
ZO-1: Zonula occludens-1
