# Supplementary material for: Histidine containing dipeptides protect epithelial and endothelial cell barriers from methylglyoxal induced injury
Source: Sci Rep. 2024 Nov 4;14:26640. doi: 10.1038/s41598-024-77891-9 (PMC11535046; doi:10.1038/s41598-024-77891-9)
Supplement: Supplementary file 1 — Supplementary Information 1. [file 41598_2024_77891_MOESM1_ESM.pptx]

## Slide 1
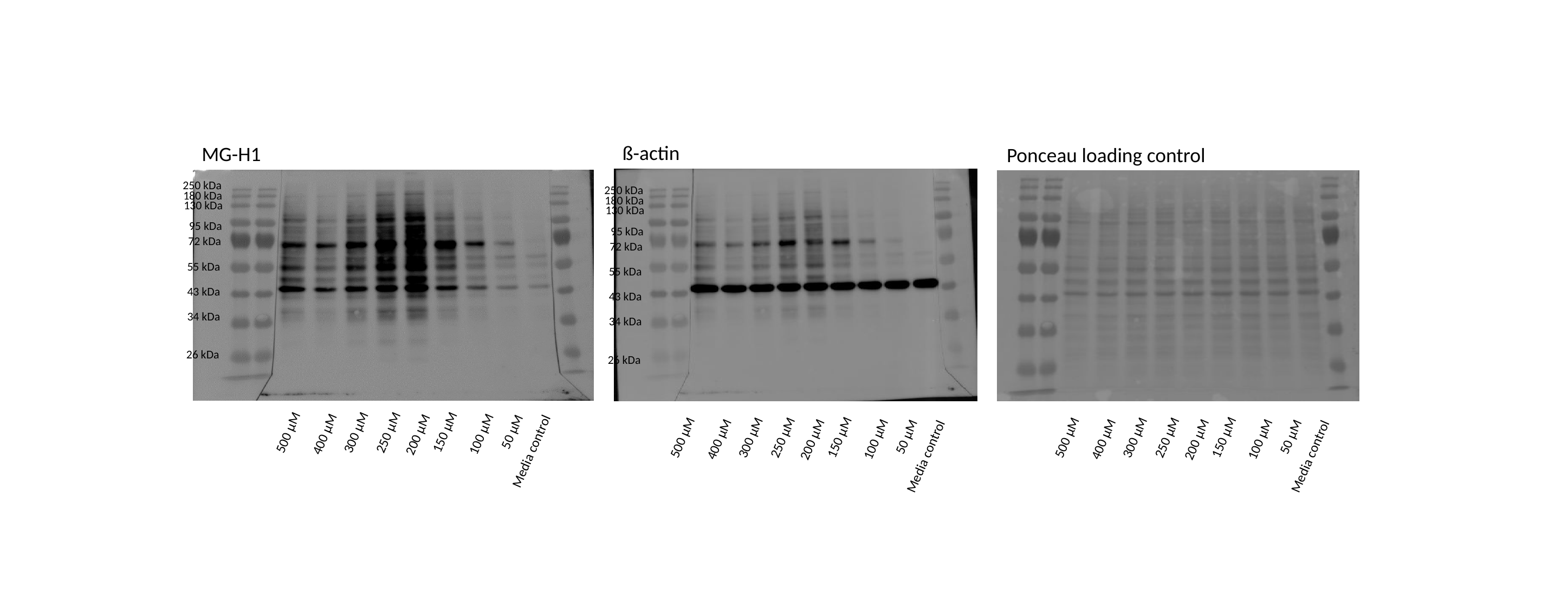

ß-actin
MG-H1
Ponceau loading control
250 kDa
250 kDa
180 kDa
180 kDa
130 kDa
130 kDa
95 kDa
95 kDa
72 kDa
72 kDa
55 kDa
55 kDa
43 kDa
43 kDa
34 kDa
34 kDa
26 kDa
26 kDa
50 µM
150 µM
500 µM
300 µM
250 µM
400 µM
100 µM
200 µM
50 µM
50 µM
150 µM
150 µM
500 µM
300 µM
250 µM
500 µM
300 µM
250 µM
400 µM
100 µM
400 µM
100 µM
200 µM
200 µM
Media control
Media control
Media control

## Slide 2
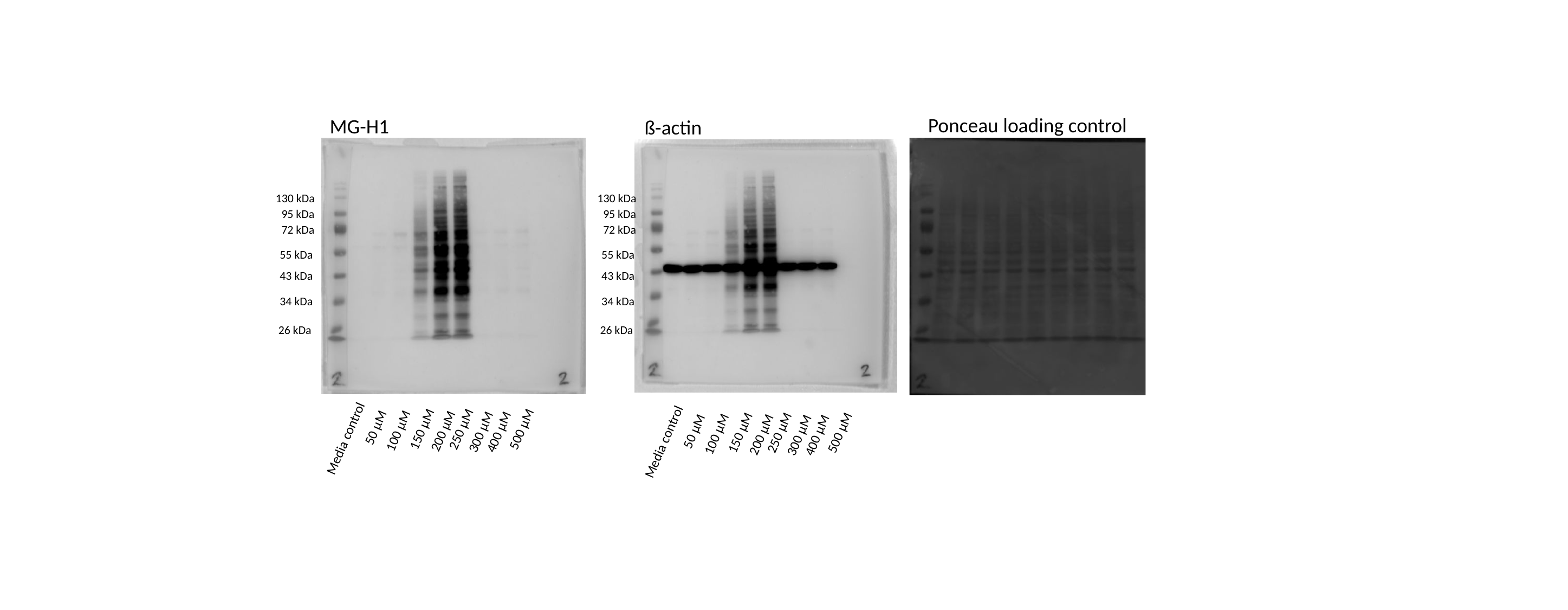

Ponceau loading control
MG-H1
ß-actin
130 kDa
130 kDa
95 kDa
95 kDa
72 kDa
72 kDa
55 kDa
55 kDa
43 kDa
43 kDa
34 kDa
34 kDa
26 kDa
26 kDa
50 µM
500 µM
250 µM
150 µM
100 µM
50 µM
200 µM
400 µM
300 µM
500 µM
250 µM
150 µM
100 µM
200 µM
400 µM
300 µM
Media control
Media control

## Slide 3
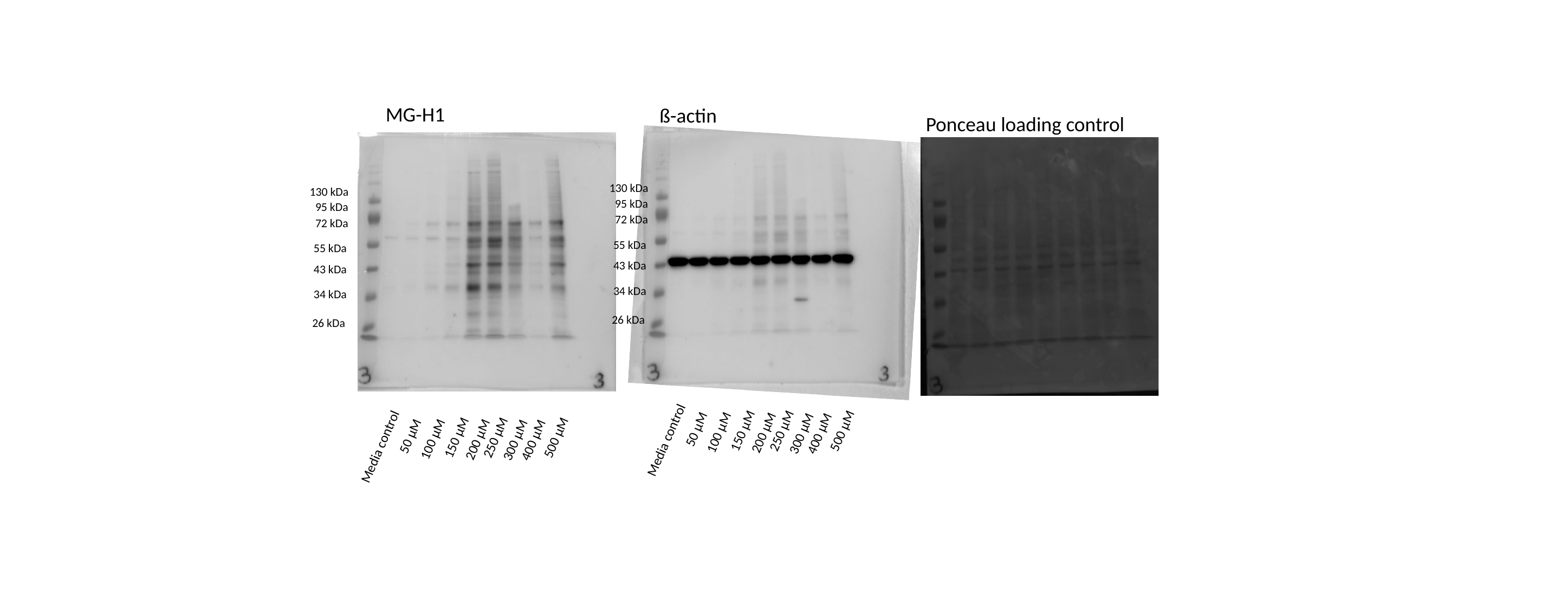

MG-H1
ß-actin
Ponceau loading control
130 kDa
130 kDa
95 kDa
95 kDa
72 kDa
72 kDa
55 kDa
55 kDa
43 kDa
43 kDa
34 kDa
34 kDa
26 kDa
26 kDa
50 µM
500 µM
250 µM
150 µM
100 µM
200 µM
400 µM
300 µM
50 µM
500 µM
250 µM
150 µM
100 µM
200 µM
Media control
400 µM
300 µM
Media control

## Slide 4
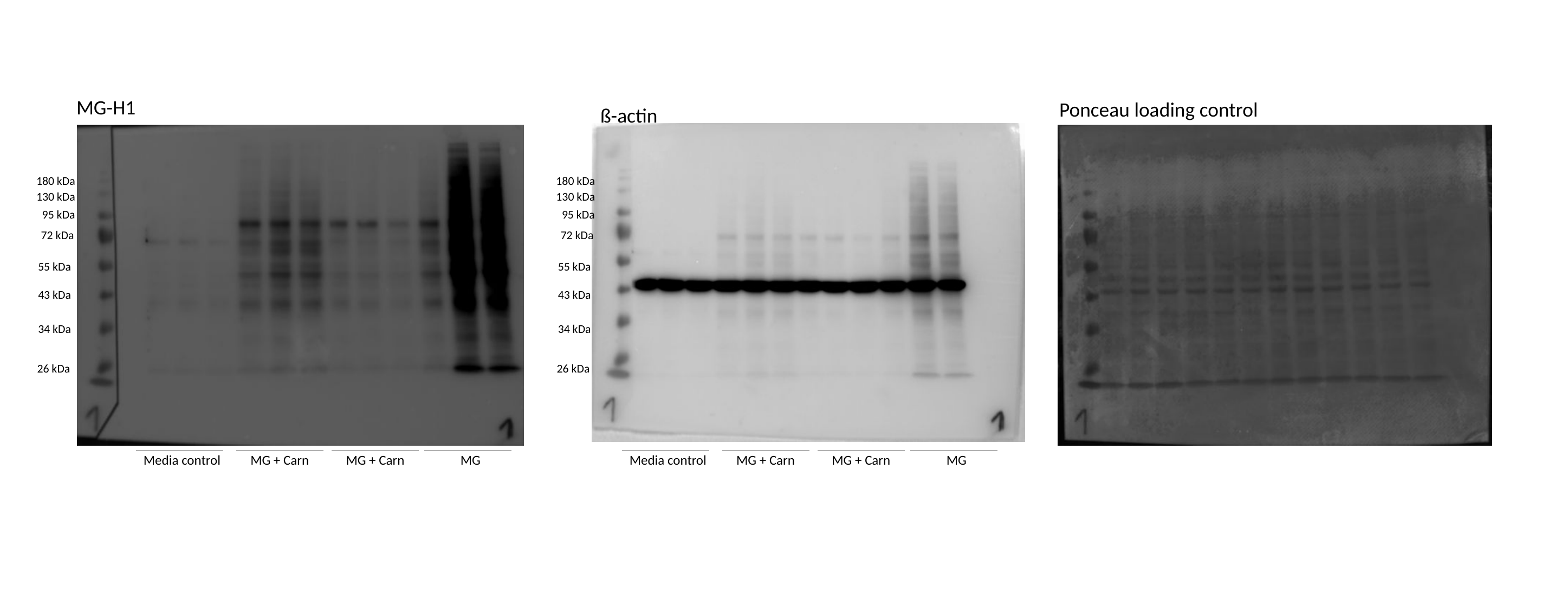

MG-H1
Ponceau loading control
ß-actin
180 kDa
180 kDa
130 kDa
130 kDa
95 kDa
95 kDa
72 kDa
72 kDa
55 kDa
55 kDa
43 kDa
43 kDa
34 kDa
34 kDa
26 kDa
26 kDa
Media control
MG + Carn
MG + Carn
MG
Media control
MG + Carn
MG + Carn
MG
