# Supplementary material for: Histidine containing dipeptides protect epithelial and endothelial cell barriers from methylglyoxal induced injury
Source: Sci Rep. 2024 Nov 4;14:26640. doi: 10.1038/s41598-024-77891-9 (PMC11535046; doi:10.1038/s41598-024-77891-9)
Supplement: Supplementary file 2 — Supplementary Information 2. [file 41598_2024_77891_MOESM2_ESM.docx]

**Supplementary Information for**

Histidine containing dipeptides protect epithelial and endothelial cell barriers from methylglyoxal induced injury

Charlotte Wetzel^1^*, Nadia Gallenstein^2^*, Verena Peters^1^*, Thomas Fleming^3,4^, Iva Marinovic^1^, Alea Bodenschatz^1^, Zhiwei Du^1^, Katharina Küper^1^, Clelia Dallanoce^5^, Giancarlo Aldini^5^, Thomas Schmoch^2,6^, Thorsten Brenner^2,7^, Markus Weigand^2^, Sotirios G. Zarogiannis^1,6^, Claus Peter Schmitt^1^, Maria Bartosova^1#^

*contributed equally

^1^Heidelberg University, Medical Faculty Heidelberg, Centre for Paediatric and Adolescent Medicine, Heidelberg, Germany

^2^Heidelberg University, Medical Faculty Heidelberg, Department of Anesthesiology, Heidelberg, Ger-many.

^3^Heidelberg University, Medical Faculty Heidelberg, Internal Medicine I and Clinical Chemistry, Heidelberg, Germany.

^4^German Center for Diabetes Research (DZD), Neuherberg, Germany

^5^Department of Pharmaceutical Sciences, Medicinal Chemistry Section "Pietro Pratesi", University of Milan, Milan, Italy

^6^Department of Anesthesiology and Intensive Care Medicine, Hôpitaux Robert Schuman – Hôpital Kirchberg, Luxembourg City, Luxembourg

^7^Department of Anesthesiology and Intensive Care Medicine, University Hospital Essen, University Duisburg-Essen, Essen, Germany.

^8^Department of Physiology, Faculty of Medicine, University of Thessaly, Larissa, Greece.

**Corresponding author**

Maria Bartosova, PhD

Division of Pediatric Nephrology

Center for Pediatric and Adolescent Medicine

Im Neuenheimer Feld 430

69120 Heidelberg, Germany

Phone +49-6221-56-35267

Fax: +49-6221-56-6487

Email: [maria.bartosova@med.uni-heidelberg.de](mailto:maria.bartosova@med.uni-heidelberg.de)

**Supplementary Figures**


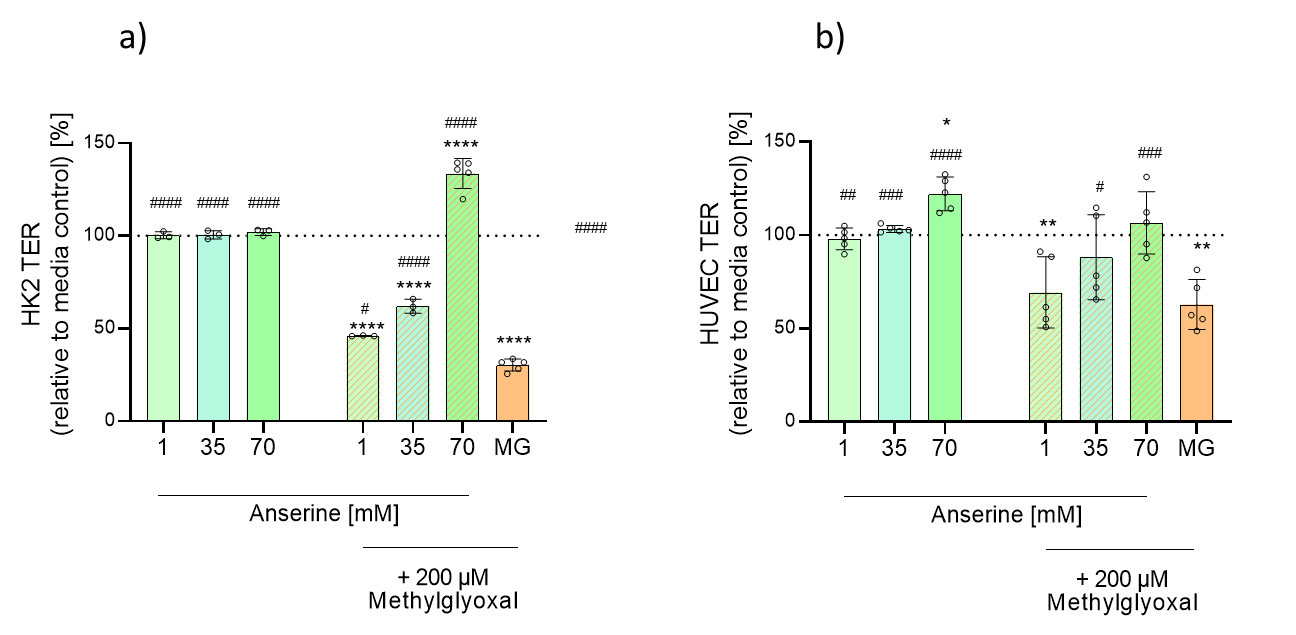


**Figure S1:** Anserin added to medium only does not impact on transepithelial resistance (TER) in human proximal tubulus cells (HK2), while in human umbilical vein endothelial cells (HUVEC) high doses of anserine increase TER. The impact on ameliorating methylglyoxal (MG, 200 µM) induced TER reduction is dose dependent in both cell types. One-way ANOVA with Tukey’s test. *: p<0.05; **: p<0.01; ***: p<0.001; ****; p<0.0001. (*compared to control, # compared to MG)


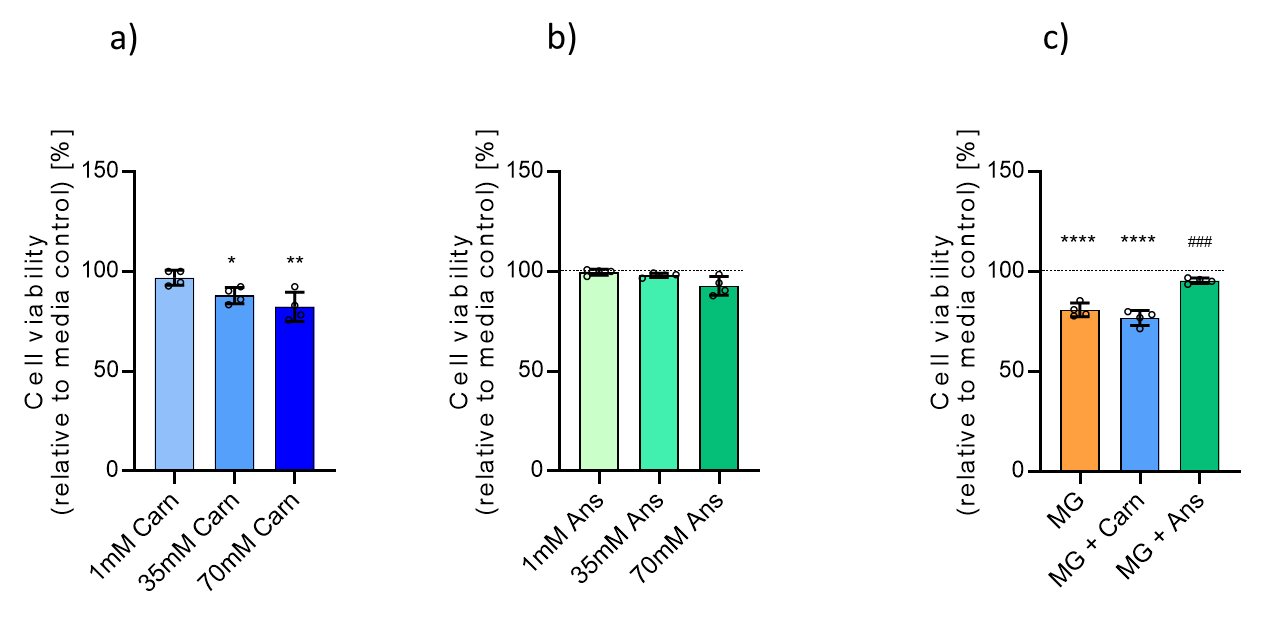


**Figure S2**: Cell viability (MTT assay) of human umbilical endothelial vein cells (HUVEC) decreases with high concentrations of carnosine (Carn, a), but not with anserine (Ans, b). Anserin but not carnosine (both 70 mM) prevents methylglyoxal (MG, 200µM) induced reduction in cell viability (c). One-way ANOVA with Tukey’s test. *: p<0.05; ***: p<0.001; ****; p<0.0001. (*compared to control, # compared to MG)

**
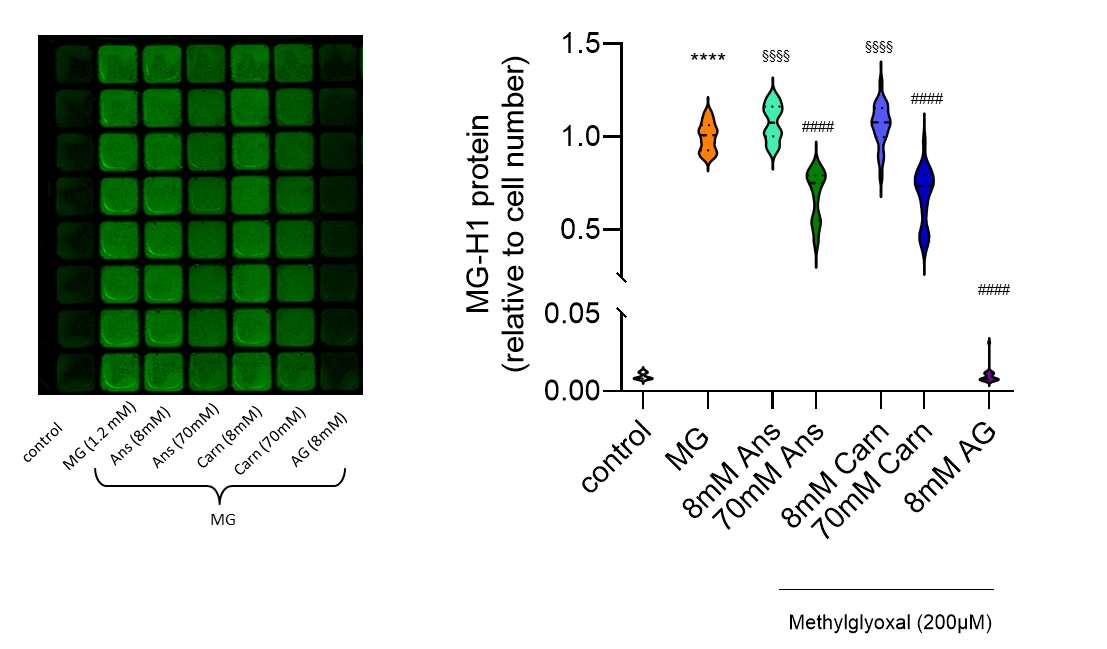
**

**Figure S3:** Aminoguanidine (AG, 8 mM) effectively quenches MGH-1 formation in HUVEC, while carnosine and anserine have no quenching capacity at comparable concentration. At higher concentrations both dipeptides anserine (Ans) and Carnosine (Carn, both 70 mM) exert some quenching activity against methylglyoxal (MG, 200 µM). One-way ANOVA followed by Sidak´s multiple comparison correction, MG was compared to medium control, all other conditions were compared to MG; Carn and Ans (both 8 mM) were compared to the same concentration of AG. ****; p<0.0001. (*compared to control, # compared to MG, § compared to AG).
